# Genome-Wide Association Study Reveals Novel Candidate Genes Associated with Productivity and Disease Resistance to *Moniliophthora* spp. in Cacao (*Theobroma cacao* L.)

**DOI:** 10.1534/g3.120.401153

**Published:** 2020-03-11

**Authors:** Jaime A. Osorio-Guarín, Jhon A. Berdugo-Cely, Roberto A. Coronado-Silva, Eliana Baez, Yeirme Jaimes, Roxana Yockteng

**Affiliations:** * Centro de Investigación Tibaitatá, Corporación Colombiana de Investigación Agropecuaria, Agrosavia, Bogotá, Colombia; † Centro de Investigación Turipaná Corporación Colombiana de Investigación Agropecuaria, Agrosavia, Montería, Colombia; ‡ Centro de Investigación La Suiza Corporación Colombiana de Investigación Agropecuaria, Agrosavia, Rionegro, Colombia; § Muséum National d’Histoire Naturelle, UMR-CNRS 7205, Paris, France

**Keywords:** Association mapping, Cacao, Genotyping-by-sequencing, Healthy pods, Monilia, Witches’ broom

## Abstract

Cacao (*Theobroma cacao* L.), the source of chocolate, is one of the most important commodity products worldwide that helps improve the economic livelihood of farmers. Diseases like frosty pod rot caused by *Moniliophthora roreri* and witches’ broom caused by *Moniliophthora perniciosa* limit the cacao productivity, this can be solved by using resistant varieties. In the current study, we sequenced 229 cacao accessions using genotyping-by-sequencing to examine the genetic diversity and population structure employing 9,003 and 8,131 single nucleotide polymorphisms recovered by mapping against two cacao genomes (Criollo B97-61/B2 v2 and Matina 1-6 v1.1). In the phenotypic evaluation, three promising accessions for productivity and 10 with good tolerance to the frosty pod rot and witches’ broom diseases were found. A genome-wide association study was performed on 102 accessions, discovering two genes associated with productivity and seven to disease resistance. The results enriched the knowledge of the genetic regions associated with important cacao traits that can have significant implications for conservation and breeding strategies like marker-assisted selection.

*Theobroma cacao* L (cacao) is an economically important perennial crop. It was originally domesticated from wild ancestors in Central America (Cuatrecasas 1964; Motamayor *et al.* 2002) and is currently produced commercially in more than 50 countries of tropical regions of Central and South America, Asia and Africa (Wickramasuriya and Dunwell 2018). Commercial cacao was initially classified into three groups based on morphological traits: Criollo, Forastero, and Trinitario (Cheesman 1944). The Criollo group shows the finest flavor but is highly susceptible to diseases (Wood and Lass 1985). The Forastero group is less susceptible to diseases, provides higher yield, and represents the most commonly grown cacao worldwide (Wood and Lass 1985). The Trinitario group is a hybrid resulting from the cross-pollination between Criollo and Forastero (Wood and Lass 1985). However, with the development of molecular markers, it is now recognized that the species *T. cacao* is composed by 10 major genetic clusters as follows: Marañon, Curaray, Criollo, Iquitos, Nanay, Contamana, Amelonado, Purús, Nacional and Guiana (Motamayor *et al.* 2008).

Cacao beans comprise the raw material of the multibillion-dollar industry that produces chocolate and is the primary income for about 6 million smallholder farmers globally (World Cocoa Foundation 2014). In Colombia, cacao cultivation occupies an area of 177 thousand hectares, and the country is classified as the tenth producer worldwide with a yield close to 400 kg/ha and a production of 54,000 metric tons of beans (Ríos *et al.* 2017). Predictions of the cocoa market estimate that the demand will continue to increase (Fountain and Hütz-Adams 2018); on the other hand, up to 40% of the cacao production is estimated to be lost annually because of diseases produced by pathogens, such as fungi and oomycetes (Bowers *et al.* 2001; Álvarez *et al.* 2014).

Among the fungal pathogens that attack cacao, some of the most important are: 1) *Moniliophthora roreri* that causes frosty pod rot disease (FPRD) commonly known as monilia that invades growing pods (Bailey and Meinhardt 2016), and 2) *Moniliophthora perniciosa* that causes witches’ broom disease (WBD); this disease produces hypertrophy and hyperplasia of distal tissue forming abnormal stems, such as a flower cushion broom and deformed branches (Meinhardt *et al.* 2008). To control the incidence of FPRD and WBD, disease control methods that include cultural practices, application of fungicides, biological control, and development of varieties with disease resistance have been attempted (Bateman *et al.* 2005; Loguercio *et al.* 2009; Jaimes and Aranzazu 2010; Acebo-Guerrero *et al.* 2012; Tirado-gallego *et al.* 2016).

The development of resistant or high-yielding varieties could be accelerated by using marker-assisted selection (MAS), which refers to the selection of individuals based on molecular markers associated with quantitative trait loci (QTL) that control defense mechanism resistance (Collard and Mackill 2008). During recent decades, many QTL for productivity (Crouzillat *et al.* 2000; Clement *et al.* 2003) and resistance to FPRD or WBD (Brown *et al.* 2005; Faleiro *et al.* 2006; Queiroz *et al.* 2003; Motilal *et al.* 2016) have been reported. Also, based on data array of single nucleotide polymorphisms (SNPs), Royaert *et al.* (2016) described genomic regions involved in WBD resistance, and McElroy *et al.* (2018) and Romero-Navarro *et al.* (2017) identified several markers related to productivity and disease resistance to *Moniliophthora* spp.

However, these studies have two disadvantages. First, the use of bi-parental populations produce a low mapping resolution because only a limited number of recombination events can be evaluated (Korte and Farlow 2013). The second disadvantage is that the use of a SNP array does not allow finding new genetic variants (Ganal *et al.* 2012). To solve these disadvantages, a method known as genotyping-by-sequencing (GBS) allows identifying thousands of SNPs (Elshire *et al.* 2011; Ganal *et al.* 2012; Romay *et al.* 2013). This method is simple, reproducible, and a considerable number of samples can be multiplexed, being suitable for population studies, germplasm characterization, and trait mapping (Davey *et al.* 2011). The analysis of GBS data in a genome-wide association study (GWAS) takes advantage of the historical recombination events accumulated over thousands of generations, resulting in a high-resolution mapping (Brachi *et al.* 2011; Xu *et al.* 2017).

Our study is the first to explore GWAS using GBS data to identify new SNP variants distributed across the genome and associate them with important agronomic traits in *T. cacao*. The aims of this work were: 1) to assess the genetic diversity and population structure; 2) to characterize Agrosavia’s diverse collection of cacao germplasm with regards to productivity and disease resistance to FPRD and WBD; 3) to identify marker-trait associations, and finally 4) to identify genomic regions that have undergone selection.

## Materials and Methods

### Plant material

A total of 229 cacao accessions from the national germplasm bank located at the research center C.I. La Suiza (7°22’12’’ N, 73°11’39’’ W) of Corporación Colombiana de Investigación Agropecuaria (Agrosavia) (previously known as Corpoica) were used (Table S1). Agrosavia s collection is a representation of the diversity of the species and conserves not only native materials but also improved materials (Osorio-Guarín *et al.* 2017). Cacao trees were planted in 1998 under an agroforestry system in lanes with a distance of 2.5 m (Arguello *et al.* 1999). Trees from the species *Cordia gerascanthus L*. and *C. alliodora* L. were distributed in lanes every 12 m to provide shade. The accessions selected were those that represented the genetic diversity of the four clusters reported by Osorio-Guarín *et al.* (2017) which were created based on a population structure analysis of a collection of 565 samples. Besides, the accessions selected in the present study span different regions of Colombia as well as of the upper Amazon region of Brazil, Peru, and Ecuador; furthermore, 22 accessions from the breeding programs of Costa Rica and Trinidad were also used.

### DNA isolation and sequencing analysis

Genomic DNA of the 229 accessions was extracted from young leaf tissue using the DNeasy Plant Mini Kit (QIAGEN, Germany). The DNA concentration and quality were estimated using a Qubit Fluorometer v2.0 (Life Technologies, Thermo Fisher Scientific Inc.) and by electrophoresis on 1% agarose. Genomic DNA was digested using the enzymes *Bsa*XI ((N)_9_AC(N)_5_CTCC(N)_10_) (New England Biolabs) and *Csp*CI ((N)_10-11CAA_(N)_5_GTGG(N)_12-13_) (New England Biolabs), with 2.0 units each according to the library preparation protocol proposed by Osorio-Guarín *et al.* (2018). Fragments between 200–300 bp were size-selected and the resulted libraries were checked on the Bioanalyzer using a High Sensitivity DNA chip (Agilent). The pooled barcoded samples were sequenced using a paired-end strategy with 100 bp in eight lanes on an Illumina HiScan SQ instrument carried out in the Molecular Genetics Laboratory at the research center C.I. Tibaitatá (4°41’45” N - 74°12’12” W) of Agrosavia.

### In silico digestion

To identify all restriction cut-site positions for the combination of the *Bsa*XI and *Csp*CI enzymes in the two reference genomes (Argout *et al.* 2010; Motamayor *et al.* 2013), we used the restrict package from the Emboss v6.5.7.0 software (Rice *et al.* 2000). The total number of fragments along the genomes were ordered per chromosome and those with size range from 200 to 700 bp were counted and plotted with the software R (R development core team 2008). The number of fragments produced with the *in silico* predictions was compared to the resulting sequenced alignments using the package genomecov from BEDtools v2.27.0 (Quinlan and Hall 2010).

### SNP discovery

Raw sequence reads were checked for quality with FastQC (Andrews *et al.* 2012), and both ends were trimmed with Trim Galore v0.5.0 (Krueger 2018). Reads presenting a Phred quality score below 25 and a sequence length below 60 bp were removed. Samples were aligned with the software BWA v0.7.17 (Li and Durbin 2009) against two genomes, the Criollo B97-61/B2 v2 genome assembly which has a size of 314 Mpb in 10 chromosomes (Argout *et al.* 2010), and the Matina 1-6 v1.1 genome that has a size of 445 Mbp in 701 scaffolds; the last material represents the most commonly cultivated type of cacao worldwide (Motamayor *et al.* 2013). SNPs were discovered using Picard Tools v2.18.9 (Broad Institute 2019) and called with the GATK software v3.8.0 (McKenna *et al.* 2010). Finally, the SNPs were filtered to maintain bi-allelic SNPs with a minimum allele frequency of 0.05 and to remove missing data using VCFtools v4.2 (Danecek *et al.* 2011).

### Genetic diversity, linkage disequilibrium, and population structure analyses

The observed (Ho) and expected heterozygosity (He) were obtained with Cervus v3.0.7 (Kalinowski *et al.* 2007). A linkage disequilibrium (LD) analysis was performed using the VCFtools v4.2 software (Danecek *et al.* 2011), applying the sliding window of 500 bp. The LD decay was established employing a loess regression generated from the plotting of pairwise LD (*r^2^*) over an intermarker genetic distance with a threshold value of 0.1.

The population structure was analyzed using the intersected SNPs identified using a vcf file of 69 fully sequenced genomes representing the 10 recognized cacao genetic groups (Motamayor *et al.* 2008) (Table S1) generated mapping those genomes against the Criollo genome and then merged with the resulting vcf of our samples. Two analyses were carried out, a principal component analysis (PCA) and the maximum likelihood on the admixture v1.3 software (Alexander *et al.* 2009). The PCA was inferred with the vcfR (Knaus and Grünwald 2017) and adegenet (Jombart and Ahmed 2011) packages in the R Software. The admixture was run on a supervised mode in which the genetic group of each reference samples was indicated (Table S1). Visualization of the results was done with the package ggplot2 on the R software.

### Phenotypic data and statistical analyses

Phenotypic data were collected for a subset of 102 accessions that presented promising data on productivity or disease resistance during previous assessments. The number of healthy and infected pods was registered weekly using two to six clones per accession during four harvest periods from September 2016 to May 2018 (Table S2).

With the resulting data, the area under the disease progress curve (AUDPC) for all harvest periods was calculated using the formula proposed by Shaner and Finney (1977):

$$AUDPC=\sum_{n-1}^{i=1}\left(\frac{y_{i+1}+y_{i}}{2}\right)\times \left(t_{i+1}+t_{i}\right)$$

Where *yi* refers to the counting of the disease in the n^th^ observation, *ti* is the time in the n^th^ observation, and *n* is the total number of observations.

The following four variables were evaluated:

1. Healthy pods (productivity).
2. AUDPC of pods infected by FPRD.
3. AUDPC of flower cushion broom caused by WBD.
4. AUDPC of deformed branches caused by WBD.

The results were compared among the accessions through an analysis of variance (ANOVA). Correlations among traits were calculated using Pearson’s correlation coefficient (*r*) at *P* ≤ 0.05. All statistical analyses were performed in the R software.

Furthermore, we conducted a principal component analysis (PCA) with the prcomp function of the R software using the AUDPC values for each genotype. Then, a cluster analysis using the Ward method and Euclidean distance was conducted in the R package factoextra using the two first components of the PCA.

### Association analysis

A GWAS was performed for healthy pods (productivity) and AUDPC of the two diseases with the Genome Association and Prediction Integrated Tool (GAPIT) software package (Lipka *et al.* 2012). The mixed linear model (MLM) was used to minimize the risk of false association by incorporating population structure data (Q) and a kinship matrix (K) with the following equation:

y=Xa+Qb+Zu+e

Where *y* is the vector for phenotypes, *a* is the vector of marker fixed effects, *b* is a vector of fixed effects, *u* is the vector of random effects (the kinship matrix), and *e* is the vector of residuals. Moreover, *X* denotes the genotypes at the marker, *Q* is the Q-matrix, and *Z* is an identity matrix.

The Q-matrix was determined previously by the admixture v1.3 software, and the K-matrix was calculated using GAPIT with the Loiselle method. The quantile-quantile plots (Q-Q plots) were constructed to validate the appropriateness of the MLM. Further, Manhattan plots were generated using the −log_10_(*_p_*) values for each trait.

### Identification of genomic regions under selection

The site frequency spectrum and the LD pattern between polymorphic sites were calculated with the SweeD (Pavlidis *et al.* 2013) and OmegaPlus software (Kim and Nielsen 2004), respectively. The grid parameter was calculated for each chromosome to have a measure of the composite likelihood ratio (CLR) (SweeD) and the ω statistics (OmegaPlus) every 10,000 bp. The flanking region was fixed from 1,000 bp to 100,000 bp. The common outliers were found using an R script that includes the GFF file annotation to identify the genes in the region under selection.

### Data availability

Table S1 contains the list of the accessions used. The phenotypic data are provided in Table S2. Table S3 contains a summary of the statistics for the sequenced data per individual. The candidate genes under positive selection per chromosome are provided in Table S4. Files S5 and S6 include the vcf files of the SNPs discovered for Criollo and Matina, respectively. Figure S1 exhibits an *in silico* analysis of the restriction enzymes. Figure S2 presents the number of sequenced reads of each accession. Figure S3 shows the correlation among traits of the entire population. Figure S4 contains the QQ-plots for each evaluated trait. The R script for the detection of common outliers in selective sweeps is available at http://pop-gen.eu/wordpress/wp-content/uploads/2013/12/combined_analysis.zip. The raw sequencing data of the reference samples were downloaded from the BioProject PRJNA486011 (Cornejo *et al.* 2018), via NCBI. Supplemental material available at figshare: https://doi.org/10.25387/g3.10028999.

## Results

### SNP calling and in silico digestion

The sequencing of libraries generated by the GBS method resulted in 1,894 Gb raw reads. The observed read depth across samples ranged from 5.5 to 182.8 when mapped against the Criollo genome and from 7.1 to 179.6 when mapped against the Matina genome (Table S3). Genotyping the 229 accessions without missing data yielded a total of 9,003 SNPs using the Criollo genome as reference, whereas 8,131 SNPs were found with the Matina genome. The distance between SNPs markers ranged from 31.1 kb (chromosome 10) to 43.3 kb (chromosome 8), with an average of 39.8 kb.

The *in silico* digestion analysis showed a substantially higher number of fragments with optimal sizes for sequencing in an Illumina system (200 to 700 bp) in the Criollo genome (118,400) compared to the Matina genome (42,849). The number of base pairs was slightly lower in Criollo (8.5 million bp) compared to the value found in Matina (9.1 million bp) (Figure S1). Finally, the number of sequenced fragments produced per accession was lower in Matina compared to those predicted *in silico* for Criollo (Figure S2).

### Genetic diversity

Values for He and Ho of the 229 cacao accessions were slightly higher with the dataset generated using the Criollo genome as a reference (Ho = 0.350 and He = 0.317) compared with the dataset generated using the Matina genome (Ho = 0.316 and He = 0.314). Medium to high levels of genetic diversity and an excess of heterozygosity were found in this collection.

### Linkage disequilibrium and population structure analyses

**Figure 1:**
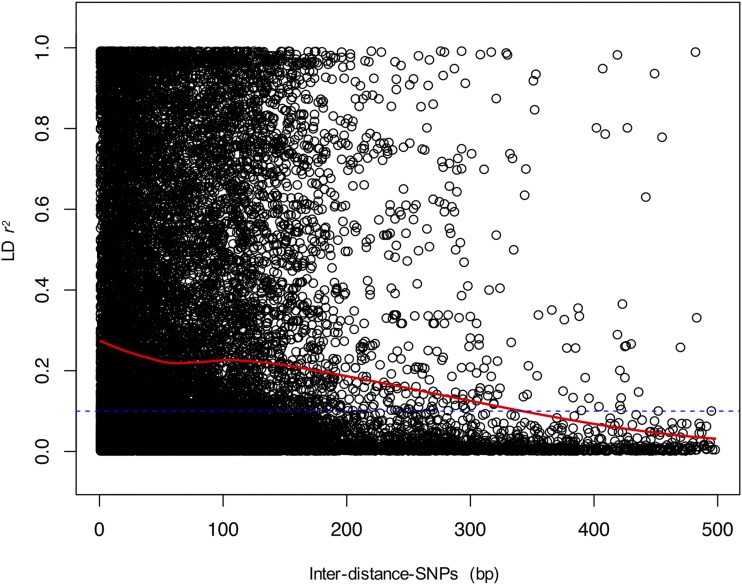
Linkage disequilibrium (LD) analysis. LD decay (*r*^2^) as a function of physical distance on all chromosomes. Only *r*^2^ values with *P* ≤ 0.05 are shown. The LD threshold of 0.1 is indicated with a blue dashed line.

The LD decay was analyzed to characterize the mapping resolution for GWAS. In the studied collection, within a sliding window of 500 bp, the LD declined quickly at an average distance of ∼320 bp (threshold of *r^2^* = 0.1) (Figure 1). The average mean *r^2^* value estimated between adjacent SNPs was 0.255.

**Figure 2:**
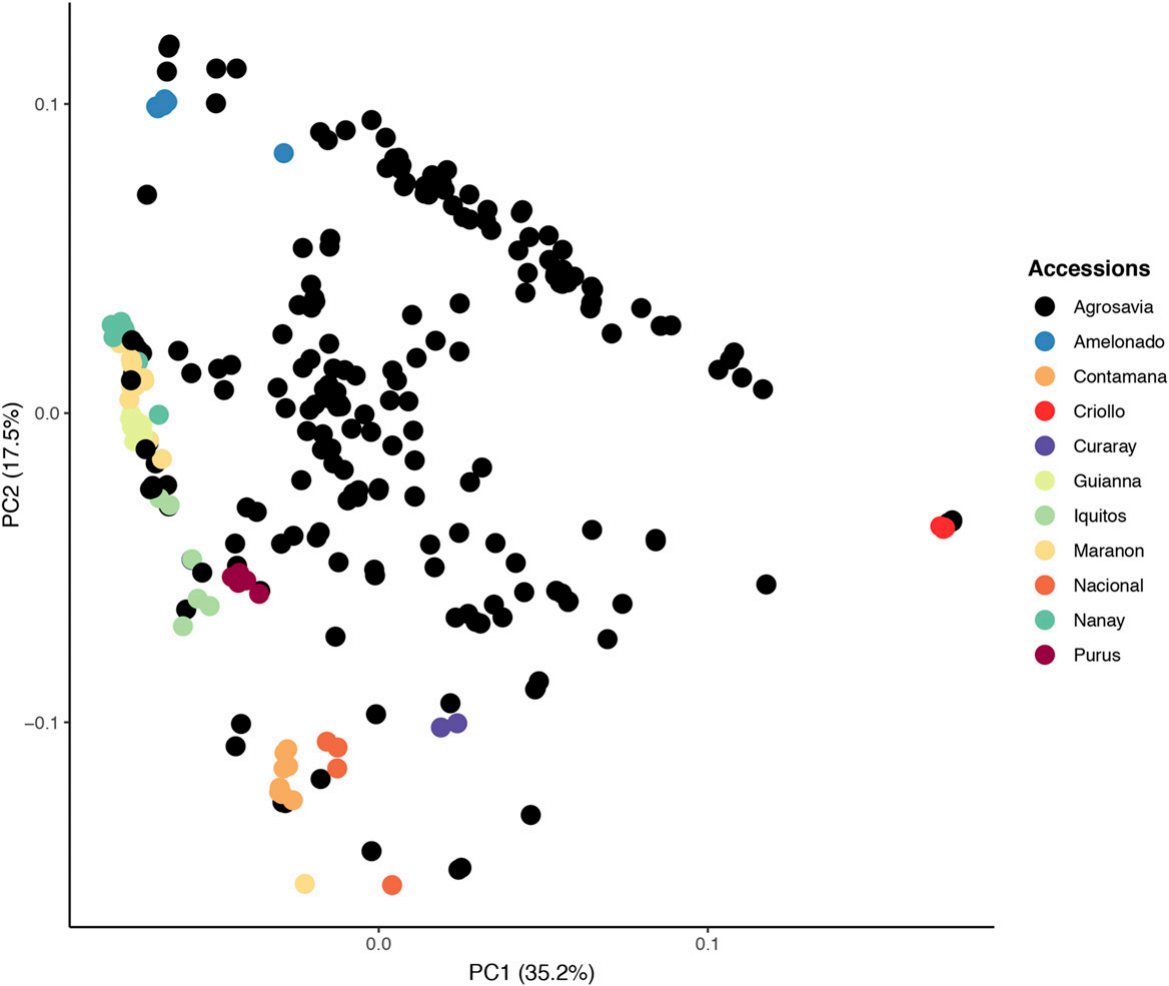
Principal component analysis (PCA) of genetic relatedness of 229 cacao accessions of the Agrosavia’s collection and 69 of the reference genetic groups, using a panel of 3,712 SNPs. Percentage of the variation captured by each component is given on the axis labels.

The analyses were carried out with 3,712 SNPs that were successfully intersected between the reference panel of 69 fully sequenced genomes and the dataset generated in this study. PCA analysis showed that the first two components provided the most information and accounted 52% of the total variation, where each component explained 35%, and 17% of the overall variation, respectively. The most differentiated group was the Criollo genetic group and the rest were not fundamentally genetically distinct from each other, as the accessions were distributed evenly among the first two PCs (Figure 2).

**Figure 3:**
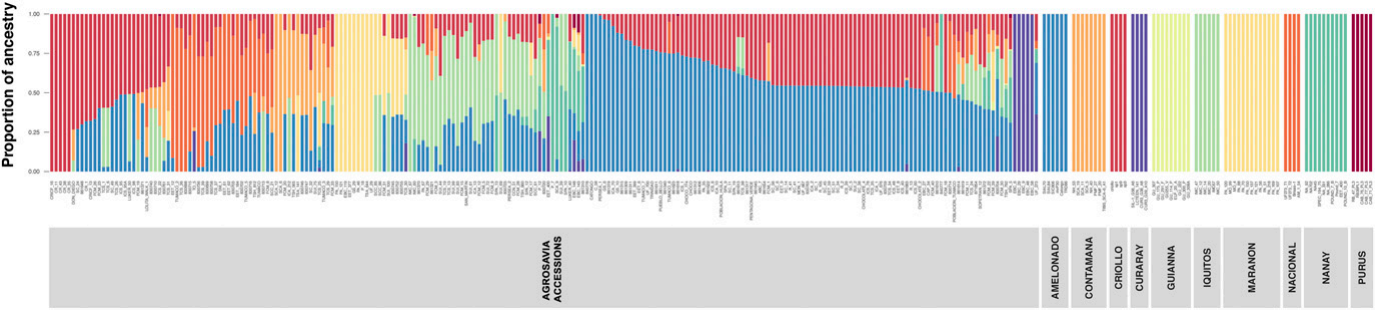
Population structure of *T. cacao* of the germplasm maintained in Agrosavia using single nucleotide polymorphisms (SNPs) mapped against the Criollo genome. The color in each bar corresponds to the probability of a genotype belonging to an assigned group. The pure clusters on right side correspond to the reference samples.

Finally, the population structure on supervised mode enabled us to identify the ancestry of Agrosavia s samples and the results were consistent with the NJ analysis result. This allowed us to confirm the ancestry of well-known accessions (*e.g.*, IMC-67 (*Iquitos*), C58 (*Amelonado*), SCA-6 (*Contamana*)), and correctly assign the ancestry of previously uncharacterized accessions (Figure 3). The results showed that the principal genetic material found in the admixed samples came from the *Amelonado* and Criollo groups.

### Phenotypic data

The cacao germplasm conserved in Agrosavia is located in a region with natural presence of inoculum of different pathogens allowing the evaluation of the disease resistance of the accessions. During the evaluation of the 102 accessions, we harvested a total of 38,178 pods with an average of 374.3 pods per plant. An imbalance between the production of healthy (6,141) and infected pods (30,477) was found. The percentage of pods that reached harvest was only 20.77%, whereas 79.23% of the pods were affected by FPRD. No pods infected by WBD were found during the harvest periods.

Correlations between phenotypic variables ranged from 0.12 to 0.54 and are shown in Figure S3. The WBD variables (flower cushion broom and deformed branches) were also positively correlated (0.54). In contrast, symptoms of FPRD and WBD diseases are weakly correlated. Finally, as expected, disease variables did not show a strong correlation with the productivity trait considered (healthy pods).

Mean, standard deviation, and coefficient of variation of the phenotypic data are shown in Table 1. The ANOVA showed significant variation between genotypes for all the analyzed traits (observed at a level of *P* ≤ 0.001), suggesting that the accessions presented in the collection are highly diverse.

**Table 1:** Means, standard deviation, coefficients of variation, and ANOVA’s test for the four tested variables

Trait	Min	Max	Mean	SD *^a^*	R^2^	CV ^b^	ANOVA p-value
**Healthy pods**	1	279	60.2	45.1	0.52	75.13	≤ 1.0^E-4^
**Pods FPRD**	14	1304	298.8	243.2	0.74	51.19	≤ 1.0^E-4^
**Flower cushion broom WBD**	0	310.5	49.3	70.5	0.73	92.28	≤ 1.0^E-4^
**Deformed branches WBD**	0	59	14.8	12.1	0.55	80.0	≤ 1.0^E-4^

a Standard deviation; ^b^ Coefficient of variation.

According to the analysis of the four harvest periods, the highest number of healthy pods was produced by the accessions GS-29 (279 pods), FCM-39 (208), and EET-8 (165) (Table S2). The genotypes CRICF-13, EBC-06, EBC-09, and SUI-72, were less affected by FPRD, as shown by the AUDPC values (Table S2). The floral cushions of genotypes EET-377, SCC-85, SCC-86, and UF-273 were less affected by WBD. The genotypes EET-377 and SCC-85 also showed the lowest number of branches affected by WBD, as well as the genotypes SUI-99 and FCM-19 (Table S2).

**Figure 4:**
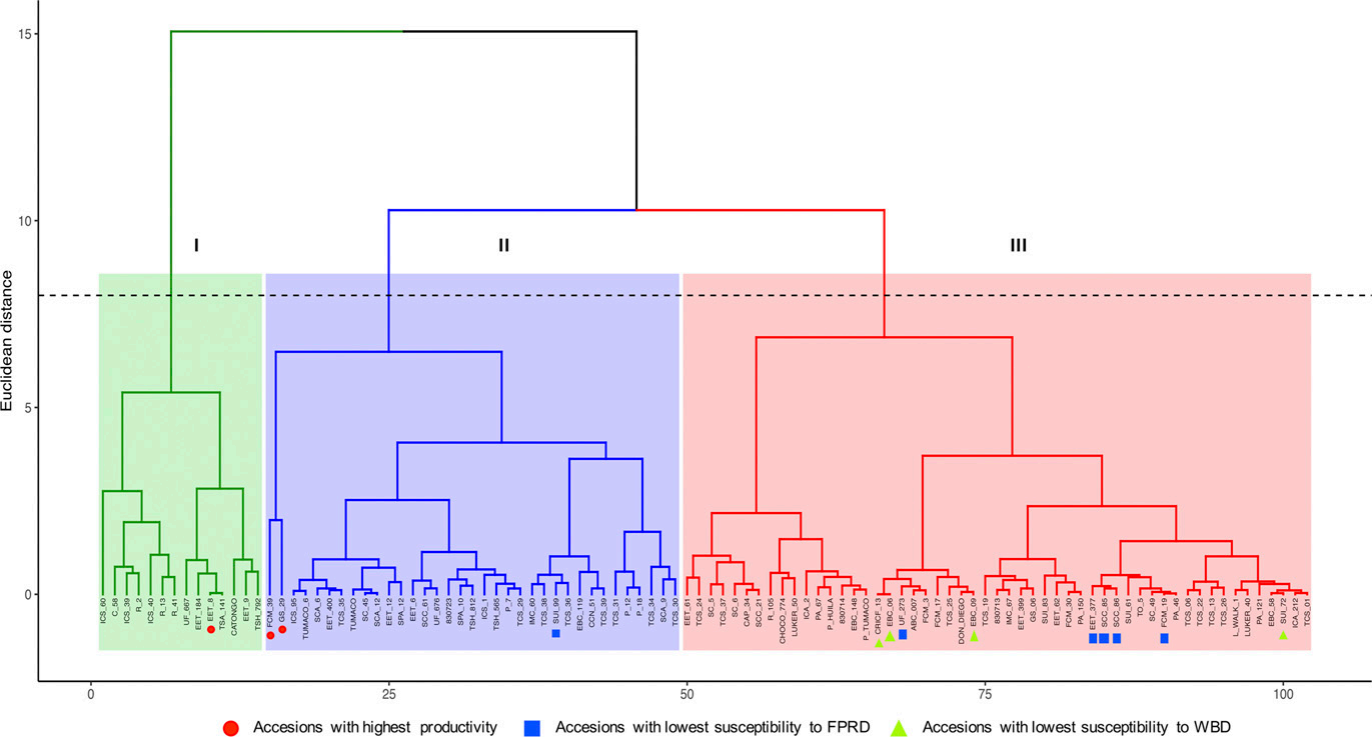
Conglomerate analysis of phenotypic data. Principal component analysis conducted using as variables the total counts of healthy pods and area under the disease progress curve (AUDPC). The best accessions for the variables evaluated are highlighted with indicators.

Based on the conglomerate analysis, the cacao accessions were divided into three main groups with different levels of productivity and susceptibility/resistance responses to WBD and FPRD (Figure 4). The first group (I) comprised 14 accessions highly susceptible to pathogens with the highest values of infected pods by FPDR and organs infected by WBD. The second group (II) included 35 accessions with higher production of healthy pods, and (III) contained 53 accessions that showed the lowest values for the traits related to diseases (Table 2).

**Table 2:** Statistics of evaluated traits for each group

Trait	Cluster	Mean	SD	CV	Min	Max
**Healthy pods**	Cluster 1	79.86	38.46	48.16	33	165
	Cluster 2	87.97	53.48	60.79	12	279
	Cluster 3	36.68	22.73	61.96	1	95
**Pods with FPRD**	Cluster 1	523.21	263.84	50.43	264.5	1304
	Cluster 2	422	253.75	60.13	116	1225.5
	Cluster 3	158.15	107.08	67.71	14	414.5
**Flower with cushion WBD**	Cluster 1	187.61	91.77	48.92	65	310.5
	Cluster 2	35.29	27.73	78.59	1	97
	Cluster 3	22.09	31.84	144.11	0	142
**Branches with WBD**	Cluster 1	32.82	14.39	43.85	8.5	59
	Cluster 2	13.33	7.05	52.93	1	37.5
	Cluster 3	11.09	9.96	89.78	0	41.5

### Association analysis

The associated SNPs were distributed in five of the 10 cacao chromosomes (Table 3). The Q-Q plots supported the association of the SNPs with the traits (*P* ≤ 0.005) and suggested that the population structure was adequately controlled in the GWAS model (Figure S4).

**Table 3:** Significant marker–trait associations for evaluated traits

Genome	Trait	SNP Position	Chromosome	p-value	FDR Adjusted p-values	Gene	SNP position relative to the candidate gene*^a^*	Candidate gene annotation
**Criollo**	**Deformed branches WBD**	9,316,991	2	2 ^E-08^	0.138	Tc02v2_g014130	0	G-type lectin S-receptor-like serine/threonine protein
		29,572,898	3	5 ^E-09^	0.182	Tc03v2_g015970	0	Protein IWS1
	**Flower cushion broom WBD**	1,064,432	2	4 ^E-07^	2 ^E-3^	Tc02v2_g001650	0	Ion channel DMI1
		770,436	2	4 ^E-07^	2 ^E-3^	Tc02v2_g001090	0	Xyloglucan galactosyltransferase
		4,523,324	2	2 ^E-07^	2 ^E-3^	Tc02v2_g007330	0	MLO-like protein
	**Pods FPRD**	18,354,976	1	2 ^E-04^	0.280	Tc00cons_t021470.1	−11.4 kb	S-locus lectin protein kinase family
	**Harvested healthy pods**	10,111,356	8	4 ^E-07^	0.330	Tc08v2_g012850	+5.2 kb	RNA-directed DNA polymerase
		21,845,071	2	7 ^E-09^	0.330	Tc00cons_t021570.1	−22,0 kb	Ty3-gypsy retrotransposon
**Matina**	**Deformed branches WBD**	9,415,422	2	6 ^E-09^	0.300	Unkown		
		27,458,385	3	1 ^E-04^	0.300	Thecc1EG015342	0	Protein IWS1
	**Flower cushion broom WBD**	1,107,779	2	5 ^E-07^	2 ^E-3^	Thecc1EG006180	0	Ion channel DMI1
		794,296	2	5 ^E-07^	2 ^E-3^	Thecc1EG006099	0	Xyloglucan galactosyl transferase
		4,567,399	2	2 ^E-08^	4 ^E-3^	Thecc1EG006939	0	MLO-like protein
	**Pods FPRD**	18,890,740	1	3 ^E-04^	0.655	Thecc1EG003068	0	Unknown
		27,025,499	1	4 ^E-04^	0.655	Thecc1EG003830	0	Retrotransposon-like protein
	**Harvested healthy pods**	9,466,348	8	1 ^E-04^	0.309	Unkown		
		8,295,774	8	3 ^E-04^	0.309			
		9,491,291	4	3 ^E-04^	0.309	Thecc1EG017982	0	Ty3-gypsy retrotransposon

a SNP position relative to the closest candidate gene: upstream and downstream SNPs of candidate genes are specified with “–” and “+,” respectively. 0 indicates that SNPs are located within the candidate gene.

**Figure 5:**
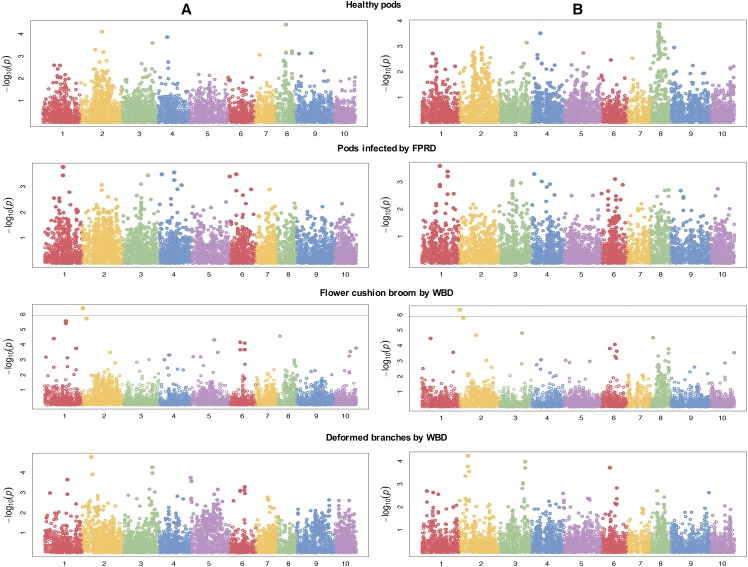
Manhattan plots of marker-trait associations for productivity, witches’ broom disease (WBD), and frosty pod rot disease (FPRD) for both reference genomes. A. Criollo. B. Matina. The green-dotted horizontal line represents the genome-wide significance threshold of *P* < 5.0 × 10^−3^.

Manhattan plots showing the log_10_(*p*)-values for the SNP markers according to their positions in each chromosome are shown in Figure 5. The GWAS identified four loci on chromosome 2 and one on chromosome 3 associated with WBD. The annotation of these loci was exactly the same using both reference genomes (Table 3).

Loci related to FPRD were located on chromosome 1 using both genomes as reference. The SNP S1_18354976 was upstream 11.4 kb to the transcript Tc00cons_t021470.1 of the gene consensus model of the Criollo genome and probably corresponded to the same SNP, *i.e.*, S1_27025499, identified with the Matina genome. A second SNP located in the gene Thecc1EG003830 was identified using the Matina genome (Table 3).

Regarding productivity, three genomic regions were identified on chromosomes 2, 4, and 8. Two candidate genes found on the Criollo genome were not located in the coding region, but they were located downstream of the gene Tc08v2_g012850 and upstream of the Tc00cons_t021570.1 transcript. Based on the Matina genome, two associated SNPs were located in chromosome 8 in genes without a functional annotation, and one SNP was located on chromosome 4 (Table 3).

### Identification of regions under selection

**Figure 6:**
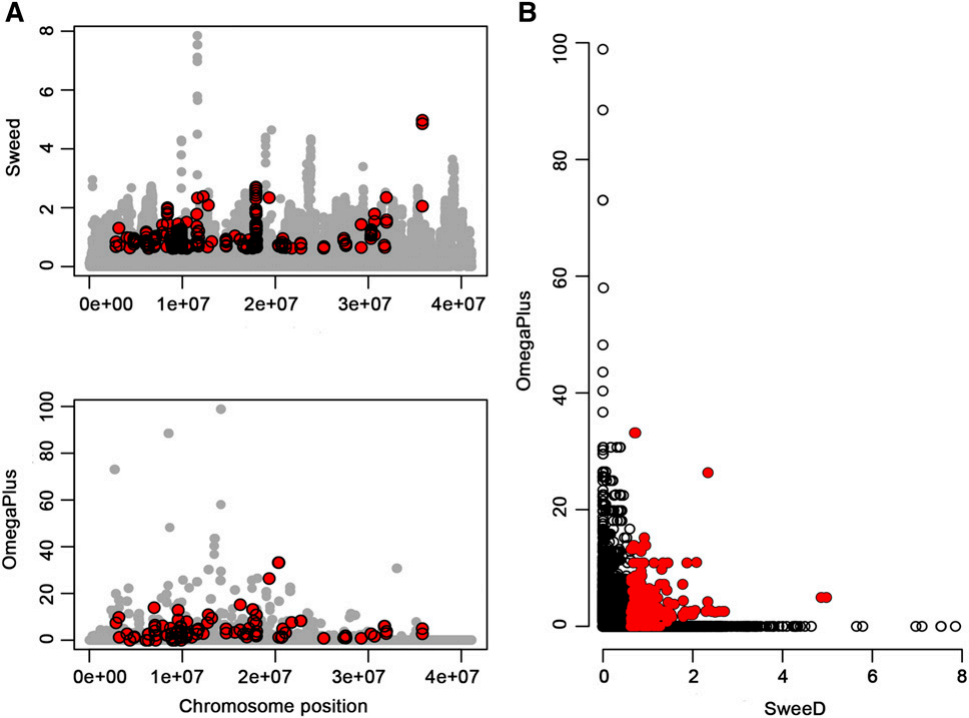
Selective sweep analysis for each chromosome. A. The x-axis denotes the position on the chromosomes, and the y-axis shows the composite likelihood ratio (CLR) evaluated with SweeD (upper panel) and the ω statistic (bottom panel) assessed with OmegaPlus. B. The joint plot for SweeD and OmegaPlus. Red dots denote outliers at a significance level of 5%.

The presence of positive selection was tested by scanning the genome for i) reduced variability regions, and ii) local patterns of high linkage disequilibrium. The highest number of genes under positive selection was identified in chromosome 3 (39), and the lowest number was found in chromosome 1 (5) (Table S4). Figure 6 showed the identified genes under positive selection throughout the genome detected by the softwares SweeD and OmegaPlus independently and the consensus of the two approaches.

The genes under selection in chromosome 1 allowed identifying a selective sweep on a region comprised between 11.1 and 22.6 million base pairs (Mbp), in which the previous associated gene Tc00cons_t021470.1 was located (Table 3). The region of chromosome 2 under selection had a size of 29.1 Mbp and is located close to the genes Tc02v2_g014130 and Tc00cons_t021570.1 associated to the response to WBD and number of healthy pods, respectively. The selective sweep on chromosome 3 covered a genomic region of 27.1 Mbp from the position 8.8 to 36 Mbp in which the gene Tc03v2_g015970 associated to the response to WBD is located. The region in chromosome 4 under selection was not related to any of the candidate genes found by the GWAS. Finally, the selective sweep for chromosome 8 included from 9.3 to 18 Mbp in which the gene Tc08v2_g012850 was associated with healthy pods.

## Discussion

The main objective of the cacao breeding program is to accumulate favorable alleles for productivity and disease resistance (Rodriguez-Medina *et al.* 2019). However, the development of an improved cacao variety could take several years because it is a perennial species with juvenile stages, which can vary from 1.5 to 3 years, depending on the genotype (de Almeida and Valle 2007). For this reason, to accelerate the selection of promising materials, it is necessary to identify genes or molecular markers associated with genomic regions involved in disease resistance or productivity.

### GBS protocol

Originally, GBS used single-enzyme digestion of frequent cuts like *Pst*I and *Apek*I (Elshire *et al.* 2011). However, this method has the disadvantage of producing a high number of short fragments and low variability in the number of reads obtained per individual (Poland *et al.* 2012). To address this issue, a study conducted by Cooke *et al.* (2016) demonstrated that the use of enzymes that cut far from the recognition site called CutSmart enzymes, like *Bsa*XI, increases the diversity of the GBS library. Osorio-Guarín *et al.* (2018) proposed a modified protocol in cacao using single-enzyme digestion with *Bsa*XI to generate a significant number of informative SNPs. In our study, we used a combination of the two enzymes (*Bsa*XI and *Csp*CI) and to confirm that this was suitable to study the cacao genome, we performed an *in silico* digestion using the two reference genomes and recovered 3 million bp more than the study of Osorio-Guarín *et al.* (2018). Moreover, we found that the combination of the two enzymes is more frequently found in the Criollo genome than in the Matina genome. The raw SNPs number in the presented study was 16,773, which is a higher value compared to the datasets obtained by Osorio-Guarín *et al.* (2018) who discovered a raw number of 12,457 SNPs in 32 samples and by Lachenaud *et al.* (2018) that reported a raw number of 9,187 SNPs using 264 samples with double-digestion using *Pst*I and *Mse*I. The results of the current study indicated the suitability of using a double-digestion for GBS libraries for cacao employing CutSmart enzymes (*Bsa*XI and *Csp*CI).

### Genotypic analysis

The genetic analysis showed that the cacao population conserved in the germplasm bank of Agrosavia has a medium to high level of genetic diversity (Ho = 0.350 and He= 0.317), that is equivalent to other studies assessing cacao diversity (Ji *et al.* 2013; Cosme *et al.* 2016; Gopaulchan *et al.* 2019; De Wever *et al.* 2019). Osorio-Guarín *et al.* (2017) found a higher heterozygosity value analyzing 565 accessions; although we used the same collection, we analyzed only 229 accessions generating lower heterozygosity results.

High-density SNP mapping facilitates understanding the genetic determinants of complex traits in GWAS. In the current study, the genome-wide LD pattern was explored using 9,003 SNPs, which provided a valuable resource for association mapping. In our association panel, the LD decay at ∼320 bp using a sliding window of 0.5 kb (threshold of *r^2^* = 0.1) indicated that the LD blocks were small, which was expected considering the out-crossing nature of the species. In contrast, Stack *et al.* (2015) identified large LD blocks with a LD decay within 5.0 - 10 Mb (threshold of *r^2^* = 0.1), possible due to the use of SSRs. The LD *r^2^* average of our study was 0.255, higher than values reported by McElroy *et al.* (2018) in the populations Ganaderia and Las Tecas (average *r^2^* values of 0.147 and 0.188, respectively), but lower compared than the population Malvinas (average *r^2^* value of 0.418). Distinct methodologies, number of markers, population sizes, genetic origins, and standard errors among the studies may account for the different results.

The population structure analyses demonstrated that the collection is diverse and has a good representation of different cacao genotypes, consistent with previous analyses (Osorio-Guarín *et al.* 2017). The spread of Agrosavia accessions in the PCA (Figure 2) is consistent with our admixture analysis (Figure 3) in which individuals are principally hybrids between Criollo and other Amazonian groups (Amelonado, Iquitos, Contamana, etc). The genetic group Criollo is the only one that is separated by the PCA analysis. This result is consistent with the study reported by (Cornejo *et al.* 2018) in which they conclude that this differentiation could be to the early diversification or recent domestication of the Criollo population from the rest of the genetic clusters. The other cacao samples do not form distinguishable groups consistent with the study of McElroy *et al.* (2018) but the distribution probably corresponds to a geographical gradient as explained by Cornejo *et al.* (2018).

The highest representation of the Amelonado and Criollo ancestries in the Agrosavia s samples (Figure 3) could be explained by the extensive use of these materials in the breeding programs to obtain high-quality flavor from Criollo genotypes combined with high yields and disease resistance from Amelonado genotypes (Wood and Lass 1985). In contrast, the Purus and Curaray genetic groups were less represented in the collection. This could be an opportunity to explore the genetic diversity of these two groups for generating new varieties in the breeding program.

### Phenotypic analysis

All the phenotypic traits evaluated in our study were positively correlated. The highest correlation (0.54) was found between flower cushion brooms and deformed branches infected both by WBD. It has been observed that *M. perniciosa* infects indiscriminately these two plant structures. In addition, low correlation was found between FPRD and WBD. Similar results were reported by McElroy *et al.* (2018) on different cacao populations evaluated. A hypothetical explanation could be that the two pathogens are competing for infecting healthy tissue. As expected, there is a low correlation between healthy pods and infected pods by the two diseases.

One important goal of this study was to identify accessions in the germplasm collection with excellent productivity or disease resistance response to FPRD and WBD. The evaluation during the four harvest periods allowed identifying three accessions with the highest productivity, GS-29 (279 pods), FCM-39 (208), and EET-8 (165) (Table S2). The accession GS-29 from Grenade was previously identified as a high productivity clone (Hunter 1990). The EET-8 accession from Ecuador was highlighted in a study conducted by Aranzazu *et al.* (2009) due to its high yield of 1,500 kg/ha, and the FCM-39 accession is part of the germplasm bank and has not be used commercially.

As expected, the incidence of FPRD was elevated because the germplasm collection is conserved at the research center La Suiza, located in the Magdalena Valley region of Colombia, considered the diversity center for *Moniliopthora roreri* (Jaimes *et al.* 2016). Four accessions, SUI-72, CRICF-13, EBC-06, and EBC-09, showed promising results of resistance against *M. roreri* (Table S2). Accession SUI-72 has additional qualities reported by Parra and Duarte (2007), such as a grain index (total grain weight per pod/number of grains per pod) of 1.73 and a pod index (number of cacao pods required to yield one pound of dry beans) of 22 that is not far from the commercial genotype CCN-51 that has the highest productivity in Colombia with a pod index of 15.2 (Jaimez *et al.* 2018). In comparison to cultivated Accessions CRICF-13, EBC-06, and EBC-09 are native Colombian genotypes not used commercially, and further studies are necessary to confirm their resistance response.

During the four harvests carried out in this research, differences were observed in the total number of healthy and infected pods. Besides, the incidence of WBD was significantly lower compared to FPRD. These results are probably because in Colombia, during 2016 and 2017, the “El Niño” phenomenon caused adverse conditions for the production of pods and the development of these diseases that require highly humid conditions and rain all year round for its fast spread and survival (Purdy and Schmidt 1996). The evaluation of the variables related to WBD allowed identifying six tolerant accessions (SCC-85, SCC-86, SUI-99, EET-377, UF-273, and FCM-19) (Table S2). The SCC genotypes (Arguello *et al.* 1999) are elite cultivars selected from regional hybrids from the department of Santander (Colombia), while accession SUI was sampled in the department of Antioquia (Colombia) and is a cacao genotype probably introduced from Central America. The EET-377 genotype has an Ecuadorian origin and is derived from the cross between EET-156 and Scavina 6 (End *et al.* 1992). The latter is a member of the Contamana ancestral group (Motamayor *et al.* 2008) and was previously identified as a resistant genotype against WBD in Brazil (Royaert *et al.* 2016). The UF-273 has beforehand been reported as a resistant genotype to FPRD (Romero-Navarro *et al.* 2017). Finally, the FCM-19 accession is part of the germplasm bank and is not used commercially.

The accessions that presented the highest values for the evaluated variables were highlighted in the conglomerate analysis (Figure 4), in which the group II present the accessions with good productivity and the group III the ones with good resistance to diseases. However, the EET-8 accession reported in our study as highly productive genotype was presented in the group I that regrouped the accessions most susceptible to diseases. This could be due to the fact that during the domestication process when selecting a characteristic of interest such as high productivity, the diversity of resistance-related genes was possibly lost (Smýkal *et al.* 2018). In fact, Yamada *et al.* (2013) found a positive correlation between number of pods and signs of infection of WBD.

Besides, the comparison of the admixture and the conglomerate analyses (Figure 3) allowed us to determine that the group I of the conglomerate analysis presented the highest percentage (31%) of Criollo ancestry, which has been reported as the group most susceptible to diseases (Albores-Flores *et al.* 2018). In contrast, the group II presented the lowest ancestry of criollo (15%) with high percentage of Amelonado type (previously classified as the Forastero genetic group) which is known for high productivity (Wood and Lass 1985). Finally, the group III has less representation of Criollo ancestry (22%) than group I and has better representation of the other groups that can explain the presence of less susceptible materials in this group III.

### Association analysis

The associations found were significant (*P* ≤ 0.05) before the FDR correction; however, only the associations for flower cushions with WBD were maintained significant after the correction. The non-significant p-values using the FDR correction is possibly due to the reduced sample size used in this study. QTL and association studies are often limited by the relatively small population sizes mapped, resulting in low statistical power and thus, rendering small- or even medium-effect QTL that are statistically non-significant and difficult to detect. Such statistically underpowered populations may also suffer from severe inflation of effect size estimates (the so-called Beavis effect) (Beavis 1998). Hence, increasing the population size and marker density is required to enable estimations that are unbiased by the Beavis effect and achieve higher statistical power (Beavis 1998; Klein 2007; Hong and Park 2012). Although in our work the marker density is high, the studied species is a perennial plant (long generation time) with limited offspring numbers, therefore to study large populations would require a considerable investment.

Bi-parental mapping studies from several F_1_ and F_2_ populations reported QTL for disease resistance to FPRD on chromosomes 1, 2, 5, 7, 8, 9 and 10 (Faleiro *et al.* 2006; Brown *et al.* 2007; Queiroz *et al.* 2003; Lanaud *et al.* 2009; McElroy *et al.* 2018) and WBD on chromosomes 1, 2, 4, 6, 7, 8, and 9 (Brown *et al.* 2005; Lanaud *et al.* 2009; McElroy *et al.* 2018). Furthermore, Royaert *et al.* (2016) identified seven candidates genes related to WBD, and Romero-Navarro *et al.* (2017) reported six genes related to FPRD. In this study, loci associated with disease resistance traits were found on the same previously reported chromosomes (*i.e.*, 1, 2, 4, 5, 6, 7, 8, and 9); however, the SNPs were located in different genomic regions (Figure 5 and Table 3).

For WBD resistance response traits, three statistically significant genes were found. The first SNP was related to a G-type lectin S-receptor-like serine/threonine protein (GsSRK) that is associated with the receptor-like protein kinases (*RLKs*) gene family. In plants, *RLKs* show important roles in pathogen resistance induced after the activation of the recognition receptors of microbe-associated molecular patterns (Singh and Zimmerli 2013). Previous studies showed that *GsSRK* exhibited plant defense function in *Nicotiana glutinosa* (Kim *et al.* 2009) and *Capsicum annuum* (Kim *et al.* 2015), and they are also co-expressed with BIR2 kinase that confers resistance to *Arabidopsis thaliana* (Blaum *et al.* 2014). The second gene associated was the *DMI1* (Doesn’t Make Infections 1) related to ion channels (Zimmermann *et al.* 1999). Studies with ion channels in species like tomato have demonstrated their possible relationship with plant defense (Zhang *et al.* 2018). The last identified gene was related to the mildew resistance Locus O (*MLO)* protein. The *MLO* is a gene family specific to plants and plays significant roles in the resistance to powdery mildew and response to a variety of abiotic stresses, plant growth, and development (Liu *et al.* 2017). Studies on *MLO* genes have demonstrated that these confer resistance to species such as *Arabidopsis* and tomato (Acevedo-Garcia *et al.* 2014). Other candidate genes were also found for WDB resistance (*IWS1* and Xyloglucan galactosyltransferase), but further studies are necessary to determine their role in plant defense.

Two candidate genes were related to resistance response against FPRD. The first one was associated with the S-locus lectin protein, a member of the *RLKs* gene family, which is related to plant immunity (Singh and Zimmerli 2013), as explained before. The second gene was related to retrotransposons, and according to a study conducted by Zervudacki *et al.* (2018), certain retrotransposons behave as an immune‐responsive gene during pathogen defense in *Arabidopsis*.

QTL for productivity have been previously reported on chromosomes 1, 2, 4, 5, 9, and 10 (Clement *et al.* 2003). Recently, McElroy *et al.* (2018) reported QTL related to the fresh weight of cacao pods on chromosomes 1, 3, 7, 8, 9, and 10. Previous studies on healthy pods did not find candidate genes related to the trait (Romero-Navarro *et al.* 2017). In this study, we found two candidate genes associated to the healthy pod trait (Tc08v2_g012850 and Tc00cons_t021570.1); however, further studies are necessary to confirm its role in cacao productivity.

### Selective sweep analysis

A selective sweep analysis was conducted to identify nucleotide variation that can be associated with beneficial traits for plant adaptation (Stephan 2010). Recent studies demonstrated that genomic regions that exhibit selection signatures are also enriched in genes associated with biologically important traits (Wen *et al.* 2015; Xie *et al.* 2015). The discover and intepretation of regions under selection depends on the biological background of a population that is affected by different factors such as the effective population size, population structure, migration, introgression, etc. (Crisci *et al.* 2013). A comprehensive study of the evolutionary history of a population should be addressed using different approaches.

Therefore, two approaches were used to investigate regions under selection. First, the SweeD software calculates the site frequency spectrum (SFS) which describes the frequency of a beneficial mutation that shifts toward a high or low frequency derived alleles (Fay and Wu 2000). The second approach uses the OmegaPlus software that recognizes a pattern of linkage disequilibrium which is expected to increase in regions flanking a selected site, but not across it (Nielsen 2005). In order to avoid false positives errors, a consensus between the results of the LD-based method (OmegaPlus) and the results of the SFS-based method (SweeD) was done.

The chromosomes 1 and 3 showed the largest regions under selection comprising 5 and 39 genes, correspondingly. These genes were involved in different biological process; in particular genes Tc01v2_g016260, Tc03v2_g026440, and Tc03v2_g006130 could have an importance for pod productivity because they have a role in the ovule development (Byzova *et al.* 1999), the control of root growth (Pinosa *et al.* 2013) and fruit dehiscence, respectively (Fulton *et al.* 2009). The detection of selective sweeps could help unravel the genetic structure of complex traits (Chen *et al.* 2016). In our study, five out of 13 genes that were associated genes with productivity and disease resistance were located in chromosome regions under positive selection, indicating that natural selection is probably occurring in this cacao collection due to the natural incidence of FPRD and WBD in the Magdalena River valley region (Álvarez *et al.* 2014). However, further studies on evolution and domestication processes are necessary to validate these hypotheses.

Our findings expand previous genetic mapping efforts and allow increasing the mapping resolution of the regions responsible for productivity and disease resistance traits. Leveraging this information could contribute to select promising accessions in juvenile stages in greenhouse conditions, and consequently, reduce breeding cycles.

## Conclusions

This research reported the first GWAS study of disease resistance trait for WBD and FPRD in cacao, based on SNPs produced by the GBS method. In total, two candidate genes related to productivity and seven related to disease resistance response to FPRD and WBD were detected. We also identified a total of 10 promising accessions that presented a degree of resistance response, and three accessions with promising productivity values. The current work provides new knowledge on genomic regions involved in the productivity and disease resistance response, which, after functional validation, could be useful in MAS for cacao breeding programs.
